# Sarcopenia is associated with decreased gray matter volume in the parietal lobe: a longitudinal cohort study

**DOI:** 10.1186/s12877-021-02581-4

**Published:** 2021-11-02

**Authors:** Ji Hee Yu, Regina E. Y. Kim, Jin-Man Jung, So Young Park, Da Young Lee, Hyun Joo Cho, Nam Hoon Kim, Hye Jin Yoo, Ji A Seo, Sin Gon Kim, Kyung Mook Choi, Sei Hyun Baik, Chol Shin, Nan Hee Kim

**Affiliations:** 1 Division of Endocrinology and Metabolism, Department of Internal Medicine, Korea University College of Medicine, Ansan, South Korea; 2 Institute of Human Genomic Study, Korea University Ansan Hospital, Korea University College of Medicine, Ansan, South Korea; 3 Psychiatry, University of Iowa, Iowa City, IA USA; 4 Department of Neurology, Korea University Ansan Hospital, Korea University College of Medicine, Ansan, South Korea

**Keywords:** Sarcopenia, Skeletal muscle, Muscle strength, Brain atrophy, Gray matter

## Abstract

**Background:**

Substantial evidence supports an association between physical activity and cognitive function. However, the role of muscle mass and function in brain structural changes is not well known. This study investigated whether sarcopenia, defined as low muscle mass and strength, accelerates brain volume atrophy.

**Methods:**

A total of 1284 participants with sarcopenic measurements and baseline and 4-year follow-up brain magnetic resonance images were recruited from the Korean Genome and Epidemiology Study. Muscle mass was represented as appendicular skeletal muscle mass divided by the body mass index. Muscle function was measured by handgrip strength. The low mass and strength groups were defined as being in the lowest quintile of each variable for one’s sex. Sarcopenia was defined as being in the lowest quintile for both muscle mass and handgrip strength.

**Results:**

Of the 1284 participants, 12·6%, 10·8%, and 5·4% were classified as the low mass, low strength, and sarcopenia groups, respectively. The adjusted mean changes of gray matter (GM) volume during 4-year follow-up period were − 9·6 mL in the control group, whereas − 11·6 mL in the other three groups (*P* < 0·001). The significantly greater atrophy in parietal GM was observed in the sarcopenia group compared with the control group. In a joint regression model, low muscle mass, but not muscle strength, was an independent factor associated with a decrease of GM volume.

**Conclusions:**

Sarcopenia is associated with parietal GM volume atrophy, in a middle-aged population. Maintaining good levels of muscle mass could be important for brain health in later adulthood.

**Supplementary Information:**

The online version contains supplementary material available at 10.1186/s12877-021-02581-4.

## Background

As both the proportion of older people and the length of life increase throughout the world, aging-related diseases are becoming a burden on public health. It is widely known that aging is accompanied by changes in body composition, primarily a reduction in lean body mass and growth in fat mass. The term *sarcopenia* refers to a syndrome characterized by serious loss of skeletal muscle mass and strength [1]. It potentially contributes to disability and various adverse health outcomes, including falls, fractures, and mortality [2].

Substantial evidence from cross-sectional studies shows an association between muscle function and brain function [3, 4] or structure [5–8]. A positive correlation was found between gait speed and total brain volume [5, 6], but no significant link has been reported between handgrip strength and total brain volume, except for some positive associations with white matter (WM) volume [7, 8]. On the other hand, few studies have examined the relationship between muscle mass and brain structure. Burns et al. reported that lean mass was positively associated with total brain volume in participants with early Alzheimer’s disease and controls without dementia, and that association was largely driven by WM volume [9]. Total gray matter (GM) volume was not associated with lean mass except in some regional areas [9, 10]. However, those were mostly cross-sectional studies with a small number of participants. No large-scale, longitudinal study has evaluated the association between brain structure and skeletal muscle mass indices that combine muscle mass and function, as suggested by guidelines from sarcopenia working groups.

In this study, we investigated whether sarcopenia, defined as low muscle mass and strength together, is associated with brain volume atrophy in a middle-aged population, along with the independent effects of low muscle mass and function on brain volume reduction.

## Methods

### Participants

All study subjects were taken from the Ansan cohort of the Korean Genome Epidemiology Study (KoGES), an ongoing population-based cohort study that began in 2001. KoGES participants have been biennially evaluated for demographics, medical illness, and medications. During the 6th and 7th examination cycles (2011–2014), brain magnetic resonance imaging (MRI) scans and handgrip strength data were acquired. Four years later, follow-up brain MRI scans were conducted during the 8th and 9th exams (2015–2018). Further details are described elsewhere [11].

In this study, we included 1905 subjects who completed baseline dual-energy X-ray absorptiometry (DEXA) with their handgrip strength tests and had baseline and follow-up brain MRI scans. The DEXA exams were performed from the 5th examination cycle (2009–2010). We excluded 621 subjects with the following conditions: 1) past history of dementia (*n* = 1), 2) any neuropsychiatric disorders, including anxiety, mood, or psychotic disorders (*n* = 6), 3) stroke (*n* = 29), 4) any cancer (*n* = 21), 5) diabetes mellitus (*n* = 553), 6) missing data (*n* = 11). After those exclusions, 1284 participants were enrolled in this study. Each participant signed an informed consent form. This study was performed according to the principles of the Declaration of Helsinki of the World Medical Association and was approved by the Institutional Review Board of Korea University Ansan Hospital.

### Assessments

#### Demographic, anthropometric, and laboratory measurements

All participants underwent physical examinations and responded to an interviewer-administered questionnaire that included physical activity-related questions. “Do you work out regularly enough to make your body sweat? How many times a week, how many hours, and what kind of exercise do you do?” was designated to determine the frequency, time and type of self-reported exercises. Regular exercise was defined as at least three times a week and 30 min per session during the previous month regardless of type of exercise. No participants have been involved in exercise-related interventions. Lifestyle characteristics, such as smoking status and alcohol consumption, were categorized as never, former, and current. Education level was categorized into primary, secondary, and college/university levels. Height was measured to the nearest 0·1 cm using a fixed wall-scale measuring device. Weight was measured to the nearest 0·1 kg using an electronic scale that was calibrated before each measurement. Body mass index (BMI) was calculated as weight in kilograms divided by height in meters squared. Waist circumference was measured to the nearest 0·5 cm in a horizontal plane at the level of the umbilicus at the end of a normal expiration.

Blood was drawn for biochemical analysis after an overnight fast. Plasma glucose, serum triglycerides, and high-density lipoprotein cholesterol levels were measured with an autoanalyzer (ADVIA 1650; Siemens, Tarrytown, NY). High-sensitive C-reactive protein (hsCRP) levels were measured by an immunoassay (ADVIA1800, Siemens, USA). Insulin was measured with an immunoradiometric assay kit (INS-IRMA Kit; BioSource, Nivelles, Belgium) using a Packard γ counter system.

#### Brain magnetic resonance imaging and z-score calculation

All 3D T1 MRI scans were acquired using a GE Signa HDxt 1·5 T MRI scanner with an 8-channel head coil. The detailed MRI protocols are described in a previous study [11]. Brain MRI images were processed through a well-established fully automated procedure, the BRAINS AutoWorkup in the BRAINSTOOLs package [12, 13]. The MRI processing starts with spatial normalization using landmark detection [14], bias-field correction with tissue classification [13], and finally segmentation using ANTs Joint Fusion [15]. Two hundred fifteen independent brain subcompartments were automatically delineated, and the volumes were measured. The high reliability of the longitudinal measurement of two-time point MRI using the BRAINS AutoWorkup previously described [11]. The subcompartments were merged into 3 tissue classes: GM, WM, and cerebrospinal fluid. All the volume measurements were extracted from an individual’s original anatomical space. GM and WM volumes were summed to obtain total brain volume.

#### Measurement of body composition and handgrip strength

Whole body composition was determined using DEXA. Total body fat mass (g) and total and regional lean mass (g) were measured using a Lunar DPX-MD + densitometer (GE Medical Systems, Madison, WI). Appendicular skeletal muscle mass (ASM, kg) was defined as the sum of the lean soft tissue mass of the arms and legs [16], and BMI-corrected ASM (ASM/BMI) was also calculated [17]. Handgrip strength was measured using a digital strength dynamometer (TKK 5401, Takei, Japan) to evaluate muscle strength. During the assessment, participants were asked to stand upright with their feet hip-width apart and arms extended straight down to the side unless they were physically limited. The dynamometer was held by the testing hand in a neutral (not flexed or extended) and comfortable position. Two trials for each hand were performed with a one-minute rest after each trial, and the values from both hands were averaged for analysis.

#### Definition of diabetes mellitus, hypertension, heart disease, and sarcopenia

Diabetes mellitus was diagnosed when the fasting plasma glucose was ≥7·0 mmol/L, 2-h plasma glucose was ≥11·1 mmol/L after a 75 g oral glucose tolerance test, or participants took anti-diabetic medication [18]. Insulin resistance was estimated using the homeostasis model of assessment for insulin resistance (HOMA-IR), which is calculated as fasting glucose (mmol/L) x fasting insulin (μU/mL) / 22·5 [19]. Hypertension (HTN) was diagnosed when the systolic or diastolic blood pressure was equal to or above 140 or 90 mmHg, respectively, or when participants took antihypertensive medications. Participants with a documented history of myocardial infarction, coronary artery disease, or congestive heart failure were considered to have heart disease.

Participants were classified into four groups according to the presence or absence of low muscle mass and function. Low mass was defined as being in the lowest same-sex quintile of ASM/BMI but not the lowest quintile of handgrip strength. Low strength was defined as the being in the lowest same-sex quintile of handgrip strength but not the lowest quintile of ASM/BMI. Sarcopenia was defined as being in the lowest same-sex quintile of both ASM/BMI and handgrip strength. Controls were defined as those not in the lowest quintile of either ASM/BMI or handgrip strength. The cutoff point for the lowest quintile of ASM/BMI was 0·776 for males and 0·507 for females, and that for handgrip strength was 31·9 kg for males and 18·8 kg for females.

### Statistical analysis

Baseline characteristics were compared among four groups classified by muscle mass and strength using one-way analysis of variance (ANOVA) with the Tukey post hoc test for numeric variables. The chi-square test was used to compare categorical variables. Non-normally distributed variables, such as hemoglobin A1c, triglycerides, HOMA-IR, hsCRP, and handgrip strength, are presented as the median with interquartile range for each group, and differences were tested after logarithmic transformation. The brain volume change was calculated by subtracting the baseline brain volume from the follow-up brain volume. We compared adjusted mean changes in total and regional brain volume according to groups classified by baseline muscle mass and strength using analysis of covariance (ANCOVA) with the Tukey post hoc test. For parietal GM volumes significantly ssociated with sarcopenia, adjusted volume changes of subcompartment were compared for each group by ANCOVA. We also investigated associations of z-scores transformed from regional brain volume changes across groups by ANCOVA after adjusting for several relevant factors. ICV, age, sex, smoking, alcohol, exercise, education, HTN, heart disease, baseline brain volume, time interval from DEXA to the first MRI scan, and time interval between the baseline and follow-up MRI scans were included as variables. Multivariate linear regression analyses were conducted to compare the effects of ASM and handgrip strength on brain volume changes. The regression models included GM volume changes as the dependent variable, and baseline ASM, handgrip strength and BMI were included separately and together, along with adjustments for the aforementioned variables. A *P* value < 0·05 was considered statistically significant. Statistical analyses were performed using SAS version 9.1 for Windows (SAS Institute Inc., Cary, NC).

## Results

### Subject characteristics

The mean age of all participants was 58·0 ± 6·0 years (range, 49 to 80), and the mean BMI was 24·5 ± 2·8 kg/m^2^. Of the 1284 subjects, 71·2% were classified as the control group, and 12·6%, 10·8%, and 5·4% were designated as the low mass, low strength, and sarcopenia groups, respectively. The clinical characteristics of the four strata of study subjects are listed in Table 1. Participants with sarcopenia were older and less likely to be current drinkers than the control group. The sarcopenia group did less regular exercise and had lower levels of education than the control group. The low mass and sarcopenia groups were more obese than the control group (BMI = 27·4 and 25·9, respectively, versus 24·0 kg/m^2^, *P* < 0·001), and had higher triglyceride, HOMA-IR, and hsCRP levels. The sarcopenia group reported more HTN and heart disease than the control group. The average period between the baseline and follow-up brain MRI scans was 4·2 ± 0·5 years, with no significant differences among the four groups.

**Table 1:** Baseline characteristics of the study subjects

		Muscle mass/function groups			
	Control	Low mass	Low strength	Sarcopenia	***p*** value^***b***^
*N* = 1284	914	162	139	69	
Age, y	57·2 ± 5·5	58·9 ± 5·5^c^	59·7 ± 6·4^c^	62·1 ± 7·0^c^	<·001
Sex, male	420 (46·0)	69 (42·6)	57 (41·0)	34 (49·3)	0·552
Smoking, current	95 (10·4)	10 (6·2)	14 (10·1)	5 (7·3)	0·404
Alcohol, current	437 (47·8)	70 (43·2)	52 (37·4)^c^	21 (30·4)^c^	0·005
Regular exercise	344 (37·7)	43 (26·5)^c^	40 (28·8)^c^	13 (18·8)^c^	<·001
Education					0·010
Primary	65 (7·1)	21 (13·0)^c^	21 (15·1)^c^	10 (14·5)	
Secondary	624 (68·4)	106 (65·4)^c^	92 (66·2)^c^	43 (62·3)	
College/university	224 (24·5)	35 (21·6)^c^	26 (18·7)^c^	16 (23·2)	
BMI, kg/m^2^	24·0 ± 2·5	27·4 ± 2·5^c^	23·6 ± 2·7	25·9 ± 2·4^c^	<·001
Waist circumference, cm	80·2 ± 8·0	87·0 ± 8·0^c^	79·0 ± 7·5	83·9 ± 7·2^c^	<·001
SBP, mm Hg	114·0 ± 14·0	116·7 ± 14·0	113·5 ± 14·8	116·5 ± 14·0	0·060
DBP, mm Hg	74·6 ± 9·6	76·8 ± 9·6^c^	73·0 ± 9·5	73·9 ± 9·2	0·005
FPG, mmol/L	5·0 ± 0·4	5·1 ± 0·4	4·9 ± 0·4^c^	5·0 ± 0·4	<·001
HbA1c, mmol/mol^a^	37 (34–39)	37 (34–39)	37 (33–39)	37 (34–38)	0·442
Triglycerides, mmol/L^a^	1·3 (0·9–1·7)	1·4 (1·1–2·0)^c^	1·3 (1·0–1·7)	1·4 (1·0–1·8)	0·023
Total cholesterol, mmol/L	5·2 ± 0·9	5·2 ± 0·9	5·1 ± 0·9	4·9 ± 1·0^c^	0·033
HDL cholesterol, mmol/L	1·3 ± 0·3	1·2 ± 0·3^c^	1·3 ± 0·3	1·2 ± 0·3	0·004
HOMA-IR^a^	1·6 (1·3–2·1)	2·0 (1·6–2·6)^c^	1·5 (1·2–1·8)	1·7 (1·5–2·3)	<·001
hsCRP, nmol/L^a^	5·0 (3·0–9·0)	8·9 (5·3–19·7)^c^	5·4 (3·7–10·9)	7·0 (3·5–12·6)	<·001
ASM, kg	17·7 ± 4·2	15·9 ± 3·7^c^	16·1 ± 3·5^c^	15·2 ± 3·3^c^	<·001
ASM/BMI	0·74 ± 0·16	0·58 ± 0·16^c^	0·69 ± 0·14^c^	0·59 ± 0·16^c^	<·001
Handgrip strength, kg^a^	29·3 (23·3–39·4)	26·0 (22·1–37·0)	18·5 (16·9–29·6)^c^	18·7 (17·5–28·4)^c^	<·001
HTN	249 (27·2)	57 (35·2)^c^	37 (26·6)	27 (39·1)^c^	0·042
Heart disease	38 (4·2)	7 (4·3)	8 (5·8)	7 (10·1)^c^	0·133
ICV, mL	1407·2 ± 133·0	1396·9 ± 133·0	1383·1 ± 138·8	1363·5 ± 134·5^c^	0·017

Data are presented as number (%), mean ± SD, or median (interquartile range)

^a^ Statistical significance was estimated after logarithmic transformation

^b^ ANOVA test of four groups and corresponding characteristics

^c^ Significantly different from control (*P* < 0·05)

*Abbreviations*: *ASM* appendicular skeletal muscle mass; *BMI* body mass index; *DBP* diastolic blood pressure; *FPG* fasting plasma glucose; *HbA1c* hemoglobin A1c; *HDL* high-density lipoprotein; *HOMA-IR* homeostasis model assessment of insulin resistance; *hsCRP* high-sensitive C-reactive protein; *HTN* hypertension; *ICV* intracranial volume; *SBP* systolic blood pressure

### Adjusted mean changes in total and regional brain volumes in groups classified by muscle mass and strength

**Fig. 1:**
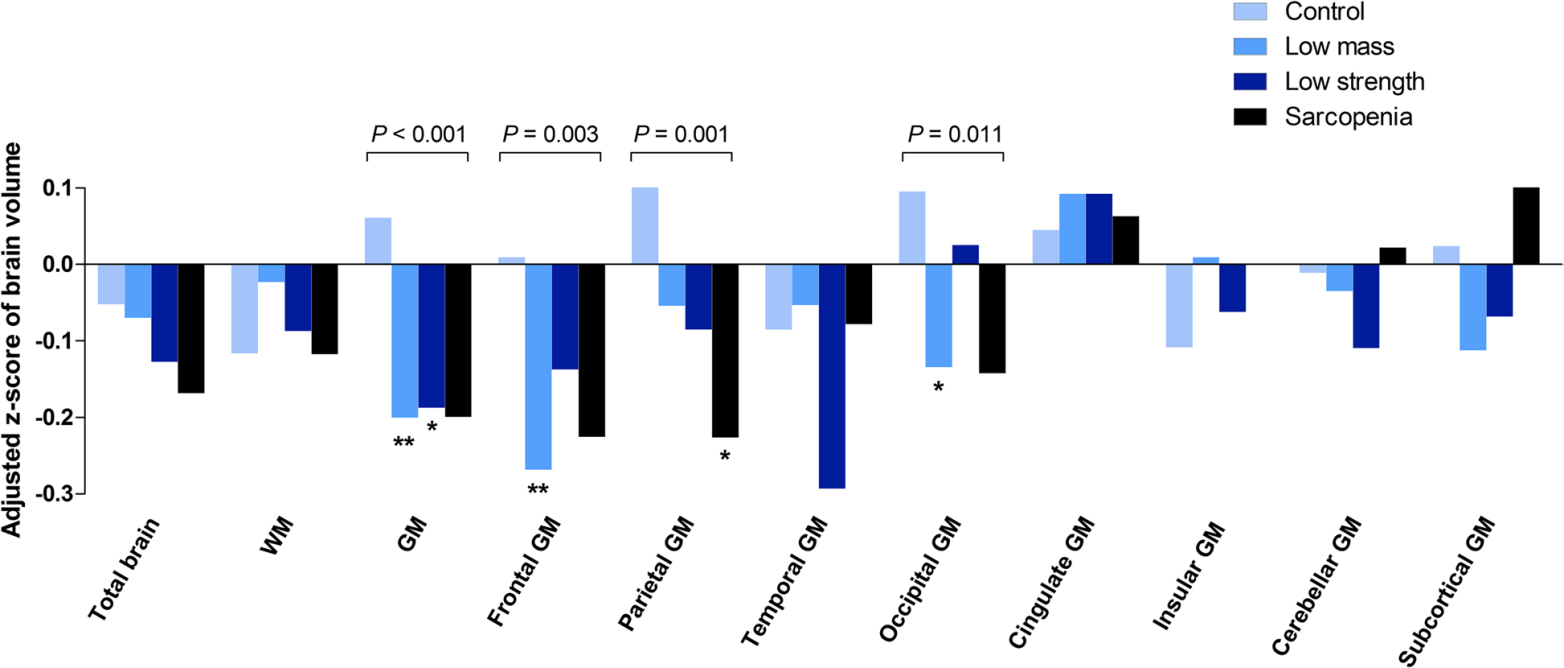
Adjusted Z-scores of longitudinal changes in total and regional brain volumes in groups classified by muscle mass and strength. ΔVolumes are adjusted for intracranial volume, age, sex, smoking, alcohol, exercise, education, hypertension, heart disease, time between dual-energy X-ray absorptiometry and first MRI, time between MRI scans, and baseline brain volume. *, Significantly (*P* < 0·05) different from control. **, Significantly (*P* < 0·01) different from control. Abbreviations: GM, gray matter; MRI, magnetic resonance imaging; WM, white matter

Table 2 and Fig. 1 present the adjusted mean changes during the four years from baseline to follow-up and the z-scores in total and regional brain volumes for the four groups categorized at baseline. The atrophy rate in GM volume differed significantly among the four groups (*P* < 0·001). The mean adjusted difference in GM volume atrophy between the control and sarcopenia group was − 2·12 mL (95% CI − 4·07 to − 0·17 mL) during 4-year follow-up period. The GM volume decrease was similarly higher in the low mass, low strength, and sarcopenia groups compared with the control, and the difference was statistically significant in the low mass and low strength groups (Fig. 1). The mean annual adjusted rate of decrease in GM volume was 0·34% per year in the control group, whereas it was 0.41, 0.42, and 0.41% per year in the low mass, low strength and sarcopenia groups, respectively (*P* = 0.009). The reduction in total brain and WM volume did not differ significantly among the four groups.

**Table 2:** Adjusted mean volume changes in total and regional brain volumes (∆ mL) during four years in groups classified by muscle mass and strength

			Muscle mass/function groups						
***∆***mL	Control		Low mass		Low strength		Sarcopenia		***p*** value^***a***^
N = 1284	914		162		139		69		
ΔTotal	−12·7	(− 15·1, − 10·3)	− 12·8	(− 16·0, −9·7)	− 14·0	(− 17·2, − 10·8)	−14·2	(− 18·2, − 10·1)	0·693
ΔWM	− 3·7	(−6·0, − 1·3)	−2·2	(−5·3, 0·9)	−3·2	(−6·4, − 0·1)	−3·9	(−7·8, 0·1)	0·663
ΔGM	− 9·6	(− 10·9, −8·3)	−11·6	(− 13·3, − 9·9)^b^	−11·6	(− 13·3, − 9·9)^b^	−11·6	(− 13·7, − 9·4)	**<·001**
ΔFrontal GM	−2·3	(− 2·9, − 1·7)	− 3·3	(− 4·1, − 2·5)^b^	− 2·8	(− 3·6, − 2·0)	−3·1	(− 4·1, − 2·1)	**0·003**
ΔParietal GM	−1·6	(− 1·9, − 1·2)	−2·0	(− 2·4, − 1·5)	−2·0	(− 2·5, − 1·6)	−2·4	(− 3·0, − 1·8)^b^	**0·002**
ΔTemporal GM	− 0·8	(− 1·2, − 0·5)	− 0·8	(− 1·3, − 0·3)	−1·4	(− 1·8, − 0·9)	−0·9	(−1·5, − 0·3)	**0·050**
ΔOccipital GM	−2·0	(− 2·4, − 1·6)	−2·5	(− 3·0, − 2·0)^b^	−2·1	(− 2·6, − 1·7)	−2·5	(− 3·1, − 1·9)	**0·016**
ΔCingulate GM	−0·4	(− 0·5, − 0·2)	−0·3	(− 0·5, − 0·1)	−0·3	(− 0·5, − 0·1)	−0·4	(− 0·7, − 0·1)	0·865
ΔInsular GM	− 0·08	(− 0·12, − 0·04)	−0·06	(− 0·11, − 0·01)	−0·08	(− 0·13, − 0·02)	−0·06	(− 0·13, 0·004)	0·682
ΔCerebellar GM	−1·5	(−1·9, − 1·2)	−1·6	(− 2·0, − 1·2)	−1·7	(− 2·2, − 1·3)	−1·5	(− 2·0, − 1·0)	0·705
ΔSubcortical GM	− 1·1	(− 1·2, − 1·0)	−1·1	(− 1·3, − 1·0)	−1·1	(− 1·3, − 1·0)	−0·9	(−1·1, − 0·8)	0·151
ΔRH.hippocampus	−0.003	(− 0.02, 0.01)	−0.008	(− 0.03, 0.01)	0.012	(− 0.01, 0.03)	−0.005	(− 0.03, 0.02)	0.221
ΔLH.hippocampus	−0.017	(−0.03, − 0.002)	−0.028	(− 0.05, − 0.01)	−0.002	(− 0.02, 0.02)	−0.014	(− 0.04, 0.01)	0.102

Data are presented as adjusted mean (95% confidence interval). Volume changes (*Δ*Volume) are adjusted for baseline intracranial volume, age, sex, smoking, alcohol, exercise, education, hypertension, heart disease, time between dual-energy X-ray absorptiometry and first MRI, time between MRI scans, and baseline brain volume. Volume changes (*Δ*Volume) were calculated by subtracting baseline brain volumes from follow-up brain volumes

^a^ ANOVA test of four groups

^b^ Significantly different from control (*P* < 0·05)

*Abbreviations*: *GM* gray matter; *MRI* magnetic resonance imaging; *WM* white matter, *RH* right hemisphere; *LH* left hemisphere

**Fig. 2:**
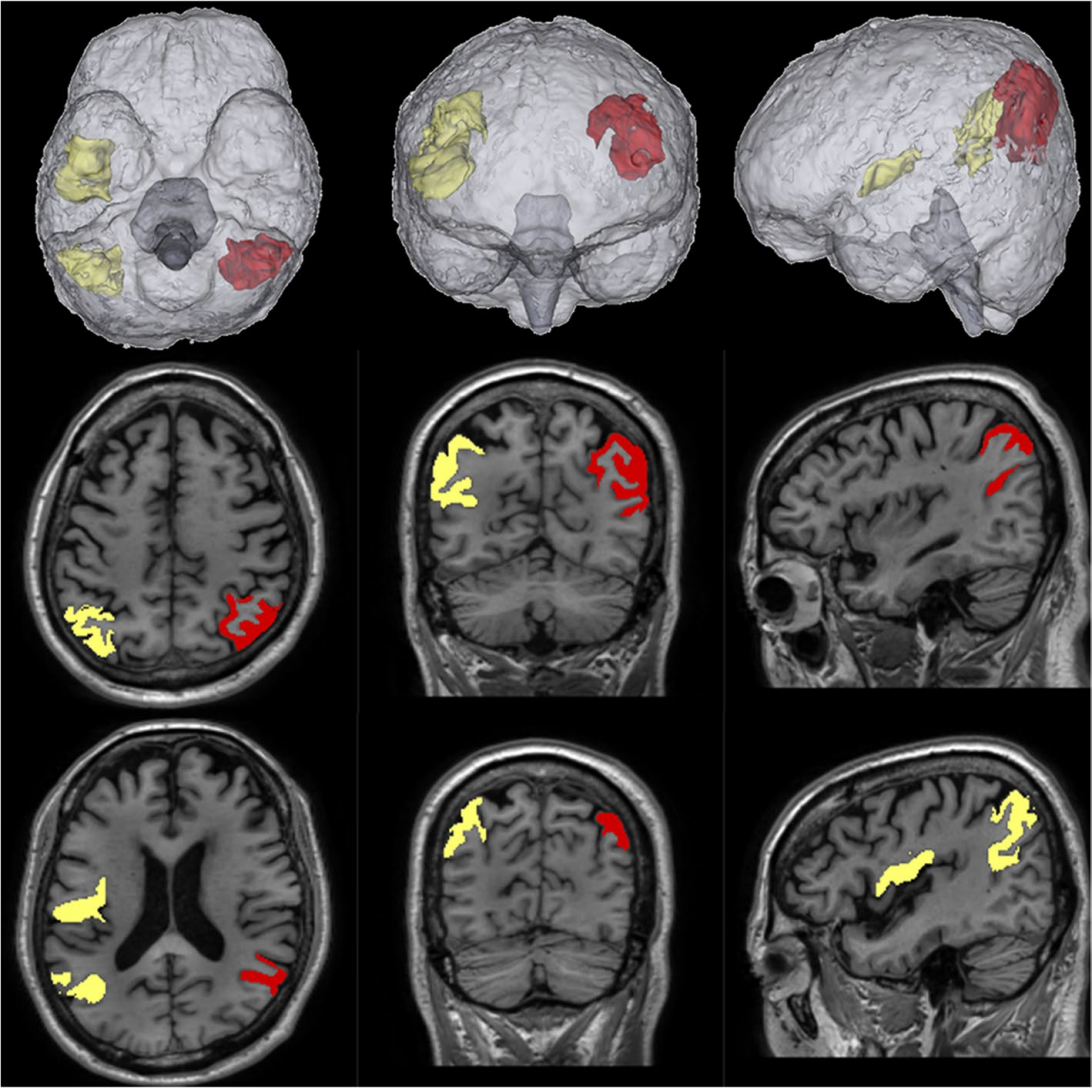
Subregions of parietal lobe with significant atrophy associated with sarcopenia. The inferior parietal lobe in the left hemisphere showed both a significant group difference (ANOVA *P* = 0·043) and a significant trend (*Trend P* = 0·044) in volume change (marked in red). The inferior lobe in the right hemisphere (yellow, *Trend P* = 0·044) showed a significant trend of faster atrophy between the control, low mass, and low strength groups and the sarcopenia group. The right supramarginal area, which showed significant group differences, is also marked in yellow (ANOVA *P* = 0·044, see Supplementary Table 1)

In the lobar brain volume analyses, the change in GM volume over four years differed significantly across groups in the frontal (*P* = 0·003), parietal (*P* = 0·002), and occipital GM (*P* = 0·016) areas (Table 2). Specifically, the low mass group showed a significantly greater decrease in the frontal and occipital GM volumes than the control group (Fig. 1). The sarcopenia group showed more prominent atrophy in the parietal GM volume than the control group (Fig. 1). The significantly greater atrophy in the parietal GM of the sarcopenia group was mostly driven by the left inferior parietal lobule (IPL) (*P* = 0·043, *Trend P* = 0·044, Fig. 2, Supplementary Table). Reductions in the temporal, cingulate, insular, cerebellar, and subcortical GM volumes did not differ among the four groups, nor did the groups differ significantly in volume changes of the hippocampus (*P* = 0.221 for ΔRH. hippocampus, *P* = 0.102 for ΔLH.hippocampus).

### Effects of baseline sarcopenic parameters on four-year GM volume changes

We evaluated the effects of muscle mass and function on GM volume changes using several regression models (Table 3). Model 1 included BMI, ASM, and handgrip strength separately with other covariates and revealed that lower ASM or handgrip strength was significantly associated with faster GM reduction (*P* = 0.048 for ASM, *P* = 0.019 for handgrip strength). Model 2 included ASM, handgrip strength, and BMI together with other covariates and showed that low muscle mass and high BMI, not muscle strength, were significantly associated with faster GM reduction (*P* = 0·034 and 0·012). The standardized regression coefficient values for the effect of ASM and handgrip strength on GM atrophy were 0·14 and 0·08, respectively (Table 3).

**Table 3:** Linear regression analyses between baseline sarcopenic parameters and GM volume changes

		β Estimate	Standardized β	SE	***p*** value
**Model 1**	1) BMI, kg/m^2^	−0·13		0·08	0·105
	2) ASM, kg	0·22		0·11	**0·048**
	3) Handgrip strength^a^, kg	2·91		1·24	**0·019**
**Model 2**	BMI, kg/m^2^	−0·22	−0·08	0.09	**0·012**
	ASM, kg	0·28	0·14	0.13	**0·034**
	Handgrip strength^a^, kg	2·06	0·08	1.33	0·123

Model 1 evaluated the effects of BMI, ASM, and handgrip strength separately in models 1), 2), and 3), respectively. Model 2 considered BMI, ASM and handgrip strength jointly. All models were adjusted for intracranial volume, age, sex, smoking, alcohol, exercise, education, hypertension, heart disease, baseline GM volume, time between dual-energy X-ray absorptiometry and first MRI, and time between MRI scans. GM volume changes were calculated by subtracting baseline brain volumes from follow-up brain volumes

^a^ Statistical significance was estimated after logarithmic transformation

*Abbreviations*: *ASM* appendicular skeletal muscle mass; *BMI* body mass index; *GM* gray matter; *MRI* magnetic resonance imaging

## Discussion

This study provides new evidence on the association between sarcopenic properties and GM atrophy in a middle-aged population. We found that low muscle mass, with or without accompanying low strength, was associated with a decrease in frontal, parietal, and occipital GM volume. The parietal lobe exhibited the highest degree of GM atrophy associated with sarcopenia, mostly driven by the left IPL. We further found that the association between muscle mass and the change in total GM volume was independent of muscle function and other covariates. To the best of our knowledge, this study is the first longitudinal study to demonstrate a relationship between parietal GM atrophy and sarcopenia, defined as reduced muscle mass and function together.

Brain volume has been observed to decline with age [20], and normal aging of the brain generally presents GM atrophy [11, 20, 21]. Among several factors affecting GM atrophy, our result provides clear evidence that it could be accelerated in individuals with low muscle mass and function. This finding is of clinical importance because GM loss has been shown to be associated with the clinical progression of dementia [22].

On the other hand, we found no significant association between muscle mass or strength and WM volume. In previous cross-sectional studies, muscle size was mainly associated with WM volume [9, 10]. Our finding could enhance the previous finding that GM volume loss starts at an earlier age and progresses more rapidly than WM volume loss [23]. A previous study reported that WM atrophy might occur in men in their 70s [11]. Another study with a longer follow-up period or with participants in their 70s and older might be required to clarify the association between sarcopenia and WM volume atrophy.

The parietal lobe, especially the left IPL exhibited the highest degree of GM atrophy associated with sarcopenia in this study. The IPL is specifically purposed to be involved in processes relating to motor attention, planning, and movement selection [24, 25]. Our findings might thus support previous neurological observations that lesions in the left hemisphere that include inferior parietal cortices often cause ideomotor apraxia [24, 26]. Moreover, GM loss in the parietal lobe has been found in the early stage of Alzheimer’s dementia and mild cognitive impairment [27], suggesting that atrophy in those areas might lead to motor and cognitive dysfunction in individuals with sarcopenia. Further research is needed to determine whether atrophy of parietal GM related to sarcopenia contributes to cognitive impairment or dementia.

Little is known about the longitudinal relationship between muscle mass and brain structural changes. We found only one follow-up study, and it concluded that a larger neck muscle cross-sectional area was associated with less whole brain atrophy [28]. Our research compared the effects of muscle mass and function on GM volume changes. In the intergroup comparison, low muscle mass and function were both associated with a greater GM volume reduction than found in the control. However, in the joint regression model, only reduced muscle mass, not muscle function, was independently associated with greater GM atrophy, and the effect size of muscle mass was also greater than that of muscle function. Although further study is required, our results suggest that muscle mass could play a greater role than muscle function in preserving GM volume with age. This is the first study to compare the effect size of muscle mass and function on future GM volume decline. Further large-scale, long-term follow-up studies that minimize the influence of muscle function or fat mass are needed to clarify the independent role of muscle mass in maintaining brain volume.

Still there has been no consensus for the gold standard criteria for sarcopenia. When evaluating the adequacy of muscle mass, the absolute ASM level has been modified to adjust for body size in different ways [29–31] because muscle mass is generally proportional to body size. Many previous studies have used ASM/height^2^ as an indicator of sarcopenia, but it shows a positive correlation with BMI and could underestimate sarcopenia in people with a high BMI [32]. ASM/BMI was proposed by the FNIH Sarcopenia Project as a relatively new operational method for calculating muscle mass [17] that provides better prognostic values for body aging [33] and mortality [34] than ASM/height^2^. In addition, the cutoff point for low muscle mass has varied in different studies. We used cutoffs of the lowest quintile for each component as suggested by a number of working groups [1, 35, 36], because of an extremely low prevalance of sarcopenia in Asians using other cutoffs [37], and the lack of outcome-based cutoff values for handgrip strength. For this reason, AWGS recommends using the lower 20th percentile of the study population as the cutoff value for low muscle mass or strength [35]. Furthermore, a recent study demonstrated that using the lowest quintile had better predictive values for mortality than using other criteria [34].

Several mechanisms have been suggested to explain the link between brain health and reduced muscle mass or function. One of them is the level of physical activity, which can modulate the release of various neurochemicals [38]. Structural brain changes after physical activity are assumed to be mediated by brain-derived neurotrophic factor (BDNF) [39]. Reduced serum BDNF concentrations were linked to a decline in hippocampal volume, whereas upregulated BDNF levels after exercise training increased hippocampal volume [40]. Other possible mediators include exercise-induced alterations in insulin-like growth factor-1 [41], homocysteine [42], irisin [43], vascular endothelial-derived growth factor [44], metabolites [45], and hormonal responses, which all mediate an active interaction between muscles and the brain through muscle–organ crosstalk [46]. In this study, low muscle mass or strength was associated with greater GM atrophy even after adjusting for exercise, suggesting the existence of other mechanisms associated with the muscle itself, not the effects of physical exercise.

Some limitations of this study should be noted. First, the assessment of muscle mass and brain MRI scans were not conducted at the same time. The average period between the DEXA and brain MRI studies was 2·8 ± 1·0 years. However, even after adjusting for those time differences, regional brain volumes differed significantly by group. Second, we did not consider cognitive function in this study. Future study is needed to determine whether muscle properties are linked to the cognitive function, with or without brain atrophy in adulthood.

Our study has several strengths. First, this is a four-year, longitudinal, population-based study with a large sample size and brain MRI imaging data. Second, muscle mass was measured using DEXA, which is the most standard method. Third, muscle function was evaluated together with muscle mass to define sarcopenia, and muscle mass standardized by body size (ASM/BMI) was used as the sarcopenia parameter, based on a recent guideline. These strengths allowed us to broden our understanding of the longitudinal effects of muscle mass and function on brain atrophy more comprehensively and concisely than any previous studies.

## Conclusions

Sarcopenia is associated with parietal GM volume atrophy in a middle-aged population. Large muscle mass is independently associated with the preservation of GM volume in this large, longitudinal follow-up study.

## Supplementary Information

**Additional file 1: Supplementary Table**. Adjusted means of regional volume changes in the parietal lobe according to groups classified by muscle mass and strength.

## Data Availability

The dataset used in this study (Ansan cohort) can be provided after review and evaluation of research plan by the Korea Centers for Disease Control and Prevention (http://www.cdc.go.kr/CDC/eng/main.jsp).

## Abbreviations

ASM: Appendicular skeletal muscle mass
BMI: Body mass index
DBP: Diastolic blood pressure
DEXA: Dual-energy X-ray absorptiometry
FPG: Fasting plasma glucose
GM: Gray matter
HbA1c: Hemoglobin A1c
HDL: High-density lipoprotein
HOMA-IR: Homeostasis model assessment of insulin resistance
hsCRP: High-sensitive C-reactive protein
HTN: Hypertension
ICV: Intracranial volume
MRI: Magnetic resonance imaging
SBP: Systolic blood pressure
WM: White matter
