# Supplementary material for: Sarcopenia is associated with decreased gray matter volume in the parietal lobe: a longitudinal cohort study
Source: BMC Geriatr. 2021 Nov 2;21:622. doi: 10.1186/s12877-021-02581-4 (PMC8565062; doi:10.1186/s12877-021-02581-4)
Supplement: Supplementary file 1 — Additional file 1: Supplementary Table. Adjusted means of regional volume changes in the parietal lobe according to groups classified by muscle mass and strength. [file 12877_2021_2581_MOESM1_ESM.doc]

**Supplementary Table. Adjusted means of regional volume changes in the parietal lobe according to groups classified by muscle mass and strength**

|  |  | | **Muscle mass/function groups** | | | | | |  |  | |
| --- | --- | --- | --- | --- | --- | --- | --- | --- | --- | --- | --- |
| **ΔμL (n=1284)** | **Control (n=914)** | | **Low mass (n=162)** | | **Low strength (n=139)** | | **Sarcopenia (n=69)** | | ***P^a^*** | | ***Trend P*** |
| **Parietal lobe, Left** |  |  |  |  |  |  |  |  |  | |  |
| △inferior parietal | -369·4 | (-456·9, -281·9) | -416·9 | (-531·4, -302·4) | -482·4 | (-600·5, -364·3) | -488·7 | (-635·3, -342·1) | **0·043** | | **0·044** |
| △parietal operculum | -87·2 | (-121·3, -53·1) | -58·1 | (-102·7, -13·5) | -78·9 | (-124·9, -32·9) | -53·5 | (-110·7, 3·6) | 0·263 | | 0·326 |
| △postcentral | -11·8 | (-81·6, 58·0) | -64·4 | (-155·7, 26·9) | -13·2 | (-107·3, 81·0) | -120·3 | (-237·2, -3·4) | 0·122 | | 0·102 |
| △precuneus | -126·4 | (-175·7, -77·2) | -116·4 | (-180·9, -52·0) | -109·4 | (-175·8, -43·0) | -177·5 | (-260·0, -94·9) | 0·439 | | 0·216 |
| △supramarginal gyrus | 35·2 | (-49·1, 119·5) | -14·6 | (-124·8, 95·7) | 30·7 | (-82·8, 144·3) | -5·4 | (-146·4, 135·6) | 0·661 | | 0·704 |
| △superior parietal | -315·5 | (-390·9, -240·0) | -392·6 | (-491·3, -294·0) | -403·8 | (-505·5, -302·0) | -328·1 | (-454·3, -201·8) | 0·058 | | 0·786 |
| △supramarginal | -79·1 | (-116·1, -42·1) | -67·4 | (-115·8, -19·1) | -91·1 | (-141·0, -41·2) | -114·2 | (-176·1, -52·3) | 0·481 | | 0·145 |
| **Parietal lobe, Right** |  |  |  |  |  |  |  |  |  | |  |
| △inferior parietal | -253·2 | (-343·6, -162·8) | -310·5 | (-428·7, -192·4) | -340·2 | (-462·1, -218·3) | -389·1 | (-540·6, -237·6) | 0·077 | | **0·044** |
| △parietal operculum | -67·7 | (-94·3, -41·1) | -51·0 | (-85·7, -16·2) | -76·8 | (-112·6, -40·9) | -55·7 | (-100·2, -11·1) | 0·478 | | 0·870 |
| △postcentral | -21·9 | (-97·3, 53·4) | -91·1 | (-189·7, 7·5) | -43·2 | (-144·8, 58·5) | -150·2 | (-276·7, -23·7) | 0·064 | | 0·063 |
| △precuneus | -140·8 | (-198·4, -83·2) | -156·7 | (-232·0, -81·3) | -153·4 | (-231·1, -75·7) | -216·7 | (-313·1, -120·2) | 0·377 | | 0·105 |
| △supramarginal gyrus | -11·5 | (-87·2, 64·2) | -56·9 | (-155·9, 42·2) | -19·9 | (-122·0, 82·3) | -132·6 | (-259·4, -5·7) | 0·154 | | 0·072 |
| △superior parietal | -332·5 | (-413·3, -251·8) | -364·8 | (-470·6, -259·1) | -404·9 | (-513·8, -296·0) | -360·8 | (-496·1, -225·6) | 0·388 | | 0·518 |
| △supramarginal | -31·9 | (-61·3, -2·5) | -15·8 | (-54·3, 22·6) | -70·8 | (-110·4, -31·1) | -27·9 | (-77·1, 21·4) | **0·044** | | 0·544 |

Data are presented as adjusted mean (95% confidence interval). Volume changes (*Δ*Volume) were calculated by subtracting the baseline brain volume from the follow-up brain volume. *Δ*Volume is adjusted for intracranial volume, age, sex, smoking, alcohol, exercise, education, hypertension, heart disease, time between dual-energy X-ray absorptiometry and first MRI, time between MRI scans, and baseline regional brain volume.

^a^ ANOVA test of four groups. Abbreviation: MRI, magnetic resonance imaging.
